# Identifying biologically interpretable transcription factor knockout targets by jointly analyzing the transcription factor knockout microarray and the ChIP-chip data

**DOI:** 10.1186/1752-0509-6-102

**Published:** 2012-08-16

**Authors:** Tzu-Hsien Yang, Wei-Sheng Wu

**Affiliations:** 1 Department of Electrical Engineering, National Cheng Kung University, Tainan, 70101, Taiwan

## Abstract

**Background:**

Transcription factor knockout microarrays (TFKMs) provide useful information about gene regulation. By using statistical methods for detecting differentially expressed genes between the gene expression microarray data of the mutant and wild type strains, the TF knockout targets of the knocked-out TF can be identified. However, the identified TF knockout targets may contain a certain amount of false positives due to the experimental noises inherent in the high-throughput microarray technology. Even if the identified TF knockout targets are true, the molecular mechanisms of how a TF regulates its TF knockout targets remain unknown by this kind of statistical approaches.

**Results:**

To solve these two problems, we developed a method to filter out the false positives in the original TF knockout targets (identified by statistical approaches) so that the biologically interpretable TF knockout targets can be extracted. Our method can further generate experimentally testable hypotheses of the molecular mechanisms of how a TF regulates its biologically interpretable TF knockout targets. The details of our method are as follows. First, a TF binding network was constructed using the ChIP-chip data deposited in the YEASTRACT database. Then for each original TF knockout target, it is said to be biologically interpretable if a path (in the TF binding network) from the knocked-out TF to this target could be identified by our path search algorithm. The identified path explains how the TF may regulate this target either directly by binding to its promoter or indirectly through intermediate TFs. After checking all the original TF knockout targets, the biologically interpretable ones could be extracted and the false positives could be filtered out. We validated the biological significance of our refined (i.e., biologically interpretable) TF knockout targets by assessing their functional enrichment, expression coherence, and the prevalence of protein-protein interactions. Our refined TF knockout targets outperform the original TF knockout targets across all measures.

**Conclusions:**

By jointly analyzing the TFKM and ChIP-chip data, our method can extract the biologically interpretable TF knockout targets by identifying paths (in the TF binding network) from the knocked-out TF to these targets. The identified paths form experimentally testable hypotheses regarding the molecular mechanisms of how a TF may regulate its knockout targets. About seven hundred hypotheses generated by our methods have been experimentally validated in the literature. Our work demonstrates that integrating different data sources is a powerful approach to study complex biological systems.

## Background

A living cell responds to physiological and environmental changes mainly by reorganization of transcriptional programs, which are regulated by transcription factors (TFs) [1-5]. TFs control the expressions of their targets in two ways. TFs either directly regulate their targets by binding to the promoters or indirectly regulate their targets by the transcriptional regulatory chains through intermediate TFs [6,7]. Thus, identifying the direct and indirect targets of TFs is very crucial for understanding the transcriptional rewiring in response to various stimuli.

A powerful high-throughput experimental technology, called the transcription factor knockout microarray (TFKM) [8], is widely used to investigate the regulatory relationships between TFs and genes. First, the genome-wide gene expression profiles between a TF knockout strain and a wild type strain are measured using microarrays. Then the differentially expressed genes between these two strains can be identified by using various statistical methods [9,10]. These genes are called the TF knockout targets because their expressions change significantly due to the knockout of the TF-encoding gene under study. In yeast, experimental data of a compendium of 269 TFKMs performed by Hu et al. [8] were released in 2007. Covering almost all known TFs in yeast, these data are the most comprehensive TF knockout experiments available for any organism and provide rich information for studying gene regulation [11]. Hu et al. [8] used an error model for identifying differentially expressed genes in their TFKMs. Later, Reimand et al. [11] applied a more sophisticated statistical method, called the moderated eBayes *t*-test [12], to Hu et al.’s TFKMs and found nine times the total TF knockout targets reported by Hu et al. They also showed that their result was more biologically meaningful than that of Hu et al. However, due to the experimental noises inherent in the high-throughput microarray technology, the TF knockout targets inferred solely from the noisy TFKMs may contain a certain amount of false positives. Even if the identified TF knockout targets are true, the molecular mechanisms of how a TF regulates its TF knockout targets remain unknown by this kind of statistical approaches. Therefore, further justifications of the identified TF knockout targets are needed before they can be used as a high quality source for gene regulation study.

Unlike Reimand et al. [11] who attacked the problem from the statistical perspective, we solved this problem from the biological perspective. It is known that TFs regulate their direct targets by binding to the targets’ promoters and regulate their indirect targets by transcriptional regulatory chains through intermediate TFs [6,7]. In this paper, we proposed a method that uses this knowledge as a biological filter for extracting biologically interpretable TF knockout targets from the original TF knockout targets identified by Reimand et al. [11].

**Figure 1:**
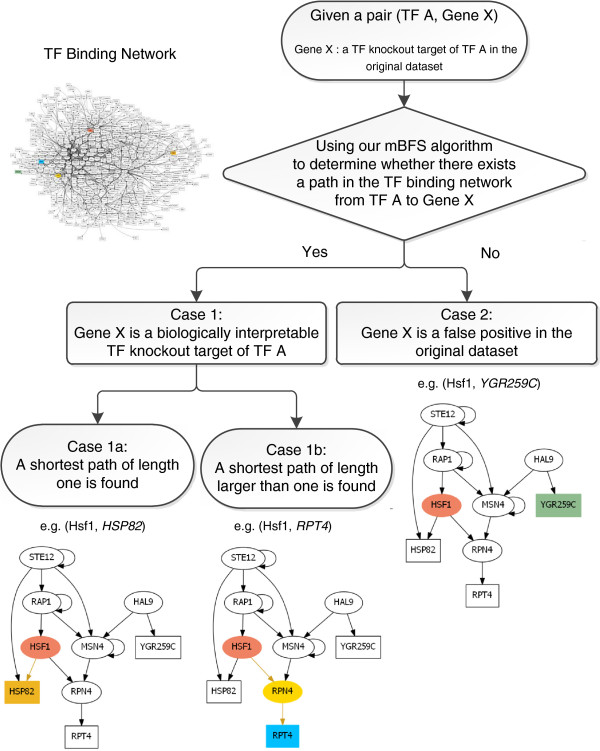
**Flowchart of our method and the examples of the outcomes.** In the TF binding network, an oval represents a TF-encoding gene and a rectangle represents a gene whose gene product is not a TF. Examples of the three possible outcomes of our method for a given TF-gene pair are as follows. In Case 1a, *HSP82* is a direct target of Hsf1. Hsf1 regulates *HSP82* by directly binding to its promoter. In Case 1b, *RPT4* is an indirect target of Hsf1. Hsf1 regulates *RPT4* through the intermediate TF *RPN4*. In Case 2, *YGR259C* is regarded as a false positive in the original dataset because no path could be found from Hsf1 to *YGR259C*.

The flowchart of our method is shown in Figure 1 and described as follows. First, a TF binding network was constructed using the ChIP-chip data, which provide experimental evidence of the binding relationships between TFs and genes. A node in the TF binding network represents a gene in the yeast genome. A directed edge from a TF-encoding gene to another gene in the TF binding network means that there exists experimental evidence (from the ChIP-chip data) showing that the TF could bind to the promoter of the gene. Then for each original TF knockout target, our modified breadth-first search (mBFS) algorithm was applied to find a shortest path from the knocked-out TF to this target in the TF binding network (see Methods for more details). There are three possible outcomes: (i) if a shortest path of length one is found, then this TF knockout target is regarded as a direct target of the knocked-out TF since the TF could bind this gene; (ii) if a shortest path of length larger than one is found, then this knockout target is regarded as an indirect target of the knocked-out TF since the TF may regulate this gene by the identified transcriptional regulatory chain through intermediate TFs; (iii) if no path could be found, then this knockout target is regarded as a false positive in the original dataset. Examples of the three possible outcomes could be seen in Figure 1. In summary, an original TF knockout target is said to be biologically interpretable if a path (in the TF binding network) from the knocked-out TF to this target could be found. The identified path might explain how the TF regulate this target either by binding to its promoter directly or by a transcriptional regulatory chain through intermediate TFs. After running this procedure, biologically interpretable TF knockout targets could be extracted from the original TF knockout targets.

## Results

### On average 90% in the original TF knockout targets are biologically interpretable

**Figure 2:**
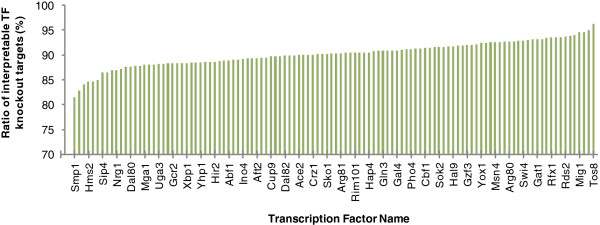
**The ratios of the biologically interpretable TF knockout targets in the original datasets.** The ratios of biologically interpretable TF knockout targets in the original datasets for the 112 TFs under study are very concentrated (mean = 0.903, standard deviation = 0.025) in a range between 0.817 (for TF Smp1) and 0.963 (for TF Tos8). In the x-axis, only 38 TF names are shown due to the space limit.

We considered 112 TFs that have enough ChIP-chip data for our analyses (see Additional file 1: Figure S1 for details). The numbers of our refined (i.e., biologically interpretable) and the original TF knockout targets identified by Reimand et al. [11] for these 112 TFs were listed in Additional file 2: Table S1. The ratios of biologically interpretable TF knockout targets in the original datasets for these 112 TFs are very concentrated (mean = 0.903, standard deviation = 0.025) in a range between 0.817 and 0.963 (see Figure 2). On average, 90% of the original TF knockout targets are biologically interpretable.

**Figure 3:**
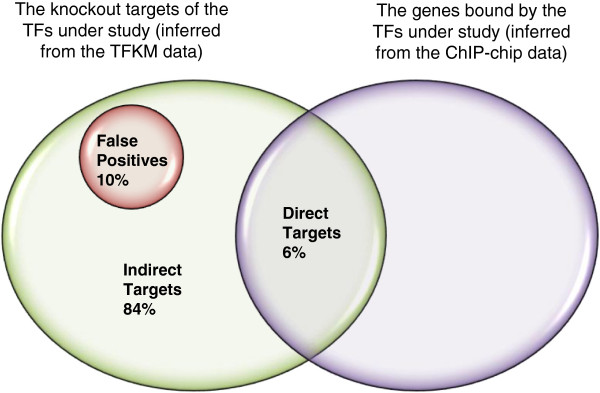
**The overlap between the TFKM data and the ChIP-chip data is very low.** Simply intersecting the TFKM data with the ChIP-chip data can only interpret 6% of the original dataset, which corresponds to those biologically interpretable TF knockout targets with the shortest paths of length one (i.e., the direct targets of the knocked-out TF). Our method can further interpret the other 84% of the original dataset, which corresponds to those biologically interpretable TF knockout targets with the shortest paths of length larger than one (i.e., the indirect targets of the knocked-out TF). The other 10% of the original dataset, for which no path could be found, is regarded as the false positives.

Note that the biologically interpretable TF knockout targets (identified by our method) cannot be found by simply intersecting the TFKM data with the ChIP-chip data since the overlap is very low (see Figure 3). This intuitive strategy can only interpret 6% of the original dataset, which corresponds to those biologically interpretable TF knockout targets with the shortest paths of length one (i.e., the direct targets of the knocked-out TF). Our method can further interpret the other 84% of the original dataset, which corresponds to those biologically interpretable TF knockout targets with the shortest paths of length larger than one (i.e., the indirect targets of the knocked-out TF).

We claim that our refined TF knockout targets are more biologically meaningful than the original ones identified by Reimand et al. [11]. To justify our claim, the following three analyses were performed.

### The refined dataset displays greater functional enrichment

Since TF knockout targets represent the genes that are co-regulated by the same TF, they should be associated with common molecular functions or biological processes. For each of the 112 TFs, the Generic GO Term Finder [13] web server was used to identify enriched GO terms [14] (with the chosen ontology aspect and FDR cutoff) in the refined and original TF knockout targets, respectively. We used all three ontology aspects (molecular function, biological process, and cellular component) and 0.05 as the FDR cutoff. Then for each TF, an enrichment score (proposed by Reimand et al. [11]) was used to measure the enrichment of functional annotations in the refined/original dataset by summing the absolute logarithms of the *p*-values of the enriched GO terms found in the refined/original dataset. Finally, an aggregate enrichment score (also proposed by Reimand et al. [11]) of the whole refined/original datasets for all 112 TFs was computed as the sum of the enrichment score for each TF.

Comparing individual TFs, the refined dataset has an equal or higher enrichment score than the original dataset in 84% (94/112) of the cases. If we compare all 112 TFs as a whole, the refined datasets also have a higher aggregate enrichment score (47859 vs. 44069) than the original datasets (see Additional file 3: Table S2 for more details). In summary, the refined dataset displays greater functional enrichment than the original dataset.

### The refined dataset has better expression coherence

Since TF knockout targets represent the genes that are co-regulated by the same TF, their expression patterns are expected to be correlated. This motivates us to test which dataset (refined or original) has higher expression coherence. The expression data were downloaded from Ihmels et al.’s study [15] which collected 1011 published genome-wide expression profiles. The testing procedure is as follows. First, two distributions were formed by computing the absolute value of the Pearson correlation coefficient between the expression data of any two genes in the refined and original dataset, respectively. Then one dataset is said to have higher expression coherence than the other if its distribution is stochastically greater than the other. The statistical significance was computed using Wilcoxon rank sum test [16]. The above procedure was applied for each of the 112 TFs under study. Finally, the *p*-values were corrected for multiple hypotheses testing to ensure FDR < 0.05.

Among 112 TFs, 55% (62/112) show significantly higher expression coherence in the refined dataset. In contrast, only 4% (4/112) show significantly higher expression coherence in the original dataset (see Additional file 4: Table S3 for more details). In summary, the refined dataset has better expression coherence than the original dataset.

### The refined dataset shows higher tendency to have physical protein-protein interactions

It has been reported that TFs tend to regulate genes that interact with each other [17]. Reimand et al. [11] proposed a measure to test this tendency by calculating the statistical significance of the TF knockout targets for being in the same protein-protein interaction module. According to Reimand et al.’s definition, a protein-protein interaction module consists of core genes and neighborhood genes. Core genes are those genes which are in the dataset and have physical protein-protein interactions with at least one gene in the dataset. Neighborhood genes are those genes which are not in the dataset but have physical protein-protein interactions with at least one of the core genes. The physical protein-protein interaction data were downloaded from BioGRID database [18]. For each of the 112 TFs, we tested whether a dataset (refined or original) is enriched in the same protein-protein interaction module using Reimand et al.’s measure. The statistical significance was computed using hypergeometric distribution [19] (see Methods for more details). Finally, the *p*-values were corrected for multiple hypotheses testing to ensure FDR < 0.05.

Of the 112 TFs, 82% (92/112) are enriched for membership to a protein-protein interaction module in the refined dataset, compared with only 71% (80/112) for the original dataset (see Additional file 5: Table S4 for more details). The refined dataset has 11% performance improvement over the original dataset on this test. In summary, the refined dataset shows higher tendency to have physical protein-protein interactions.

## Discussion

### Our method can generate experimentally testable hypotheses of how a TF may regulate its knockout targets

In our method, an original TF knockout target (identified by Reimand et al. [11]) is said to be biologically interpretable if a path from the knocked-out TF to this target could be identified in the TF binding network. The identified paths form experimentally testable hypotheses regarding the molecular mechanisms of how a TF may regulate its TF knockout targets, providing possible insights for biologists to do more detailed investigations.

**Figure 4:**
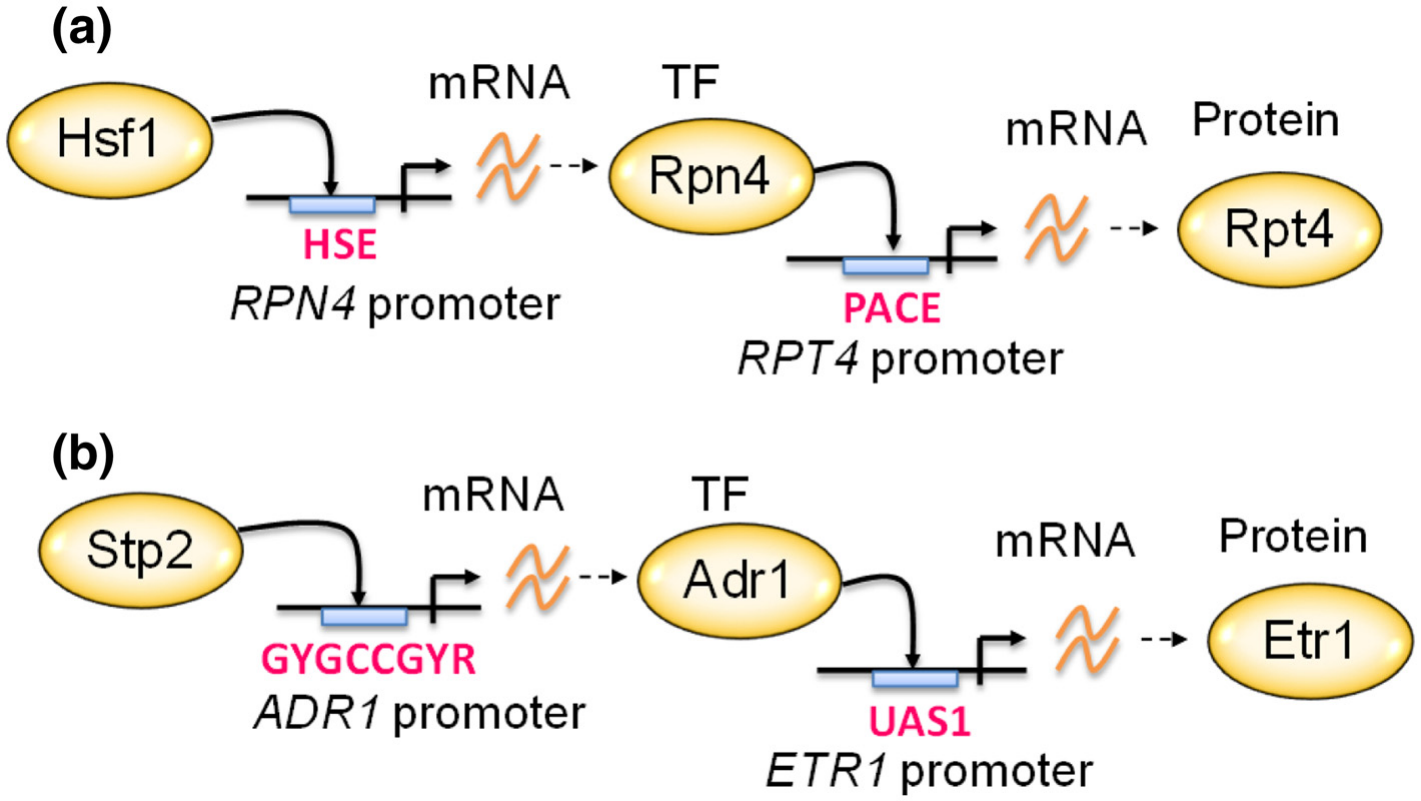
**Examples of our hypotheses that have been experimentally validated in the literature.** (**a**) *RPT4* is a knockout target of Hsf1 from Reimand et al.’s study [11]. Since the *RPT4* promoter has no Hsf1 binding sites, it is hard to imagine how Hsf1 regulates *RPT4*. Our method identified a path Hsf1→*RPN4*→*RPT4*, suggesting Hsf1 regulates *RPT4* through the intermediate TF Rpn4. The identified path has been experimentally proven to exist in the yeast cells [6,7,21-26]. (**b**) *ETR1* is a knockout target of Stp2 from Reimand et al.’s study [11]. Since the *ETR1* promoter has no Stp2 binding sites, it is hard to imagine how Stp2 regulate *ETR1*. Our method identified a path Stp2→*ADR1*→*ETR1*, suggesting Stp2 regulates *ETR1* through the intermediate TF Adr1. The identified path has been experimentally proven to exist in the yeast cells [6,7,23,27-29].

The experimentally testable hypothesis for each biologically interpretable TF knockout target could be found in Additional file 2: Table S1. About seven hundred hypotheses generated by our method have been experimentally validated in the literature (see Additional file 6: Table S5 for more details). Two examples are discussed in details here. The first example is a hypothesis of how Hsf1 regulates *RPT4*. *RPT4* is a knockout target of Hsf1 from Reimand et al.’s study [11]. *RPT4* encodes an ATPase of the 19S regulatory particle of the 26S proteasome involved in the protein degradation process and Hsf1 is a heat shock transcription factor [20]. Since the *RPT4* promoter has no Hsf1 binding sites, it is hard to imagine how Hsf1 regulates *RPT4*. Our method identified a path Hsf1→*RPN4*→*RPT4*, suggesting Hsf1 regulates *RPT4* through the intermediate TF Rpn4, a TF that regulates expression of proteasome genes involved in the protein degradation process [20]. The identified path has been experimentally proven to exist in the yeast cells. Several studies [6,7,21-26] showed that Hsf1 can directly regulate *RPN4* by binding to the HSE (heat shock element) in the *RPN4* promoter and Rpn4 can directly regulate *RPT4* by binding to the PACE (proteasome-associated control element) in the *RPT4* promoter (see Figure 4a). The heat-induced expression of Rpn4 protein (activated by Hsf1) leads to expression of Rpn4 direct targets (e.g., *RPT4*) at later stages of heat stress, providing a temporal controlling mechanism for proteasome synthesis to degrade the irreversibly damaged proteins caused by heat stress [22]. In summary, the identified path explains how a heat shock TF can regulate a protein involved in the protein degradation, indicating a close linkage between the heat shock response and the protein degradation process.

Another example is a hypothesis of how Stp2 regulates *ETR1*. *ETR1* encodes a member of the medium chain dehydrogenase/reductase family with 2-enoyl thioester reductase activity and has a probable role in fatty acid synthesis [20]. Stp2 is a TF which activates transcription of amino acid permease genes [20]. Since the *ETR1* promoter has no Stp2 binding sites, it is hard to imagine how Stp2 regulate *ETR1*. Our method identified a path Stp2→*ADR1*→*ETR1*, suggesting Stp2 regulates *ETR1* through the intermediate TF Adr1, a TF that regulates expression of genes involved in the fatty acid utilization [20]. The identified path has been experimentally proven to exist in the yeast cells. Several studies [6,7,23,27-29] showed that Stp2 can directly regulate *ADR1* by binding to the Stp2 binding site (GYGCCGYR) in the *ADR1* promoter and Adr1 can directly regulate *ETR1* by binding to the UAS1 (type 1 upstream activation sequence) in the *ETR1* promoter (see Figure 4b). In summary, the identified path explains how a TF, which activates transcription of amino acid permease genes, can regulate a protein involved in the fatty acid synthesis, indicating a close linkage between the extracellular amino acid uptake and fatty acid synthesis. Another 690 examples which also have been experimentally validated in the literature are listed in Additional file 6: Table S5.

### Our method can separate signals from noises in the original dataset

Our method classified the original TF knockout targets into biologically interpretable and uninterpretable ones. We called the former “signals” and the latter “noises”. To justify our claim, we need to prove that the signals are more biologically meaningful than the noises. We assessed the functional enrichment, the expression coherence, and the prevalence of protein-protein interactions. As shown in Table 1, the signals outperform the noises across all measures, indicating that our method is effective in separating signals from the noises in the original TF knockout targets (see Additional file 7: Table S6 for more details).

**Table 1:** The signals are more biologically meaningful than the noises

**Test (with FDR = 0.05)**	**Test results**
Functional enrichment	Our result has an equal or higher enrichment score than the noises in 94% (105/112) of the cases.
Expression coherence	Of the 112 TFs, 57% (64/112) show significantly higher expression coherence in the signals, compared with only 6% (7/112) in the noises.
The prevalence of protein-protein interactions	Of the 112 TFs, 82% (92/112) are enriched for membership to a protein-protein interaction module in the signals, compared with only 19% (21/112) in the noises

To show that the signals are more biologically meaningful than the noises, we assessed the functional enrichment, the expression coherence, and the prevalence of protein-protein interactions. The signals outperform the noises across all measures.

### Our result is better than the random results

**Figure 5:**
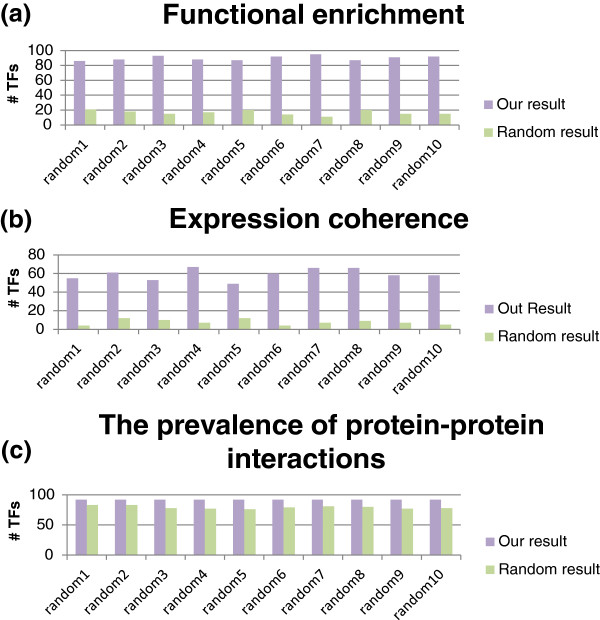
**Our result is better than the random results.** To show that our result is more biologically meaningful than the random results, we assessed the functional enrichment, the expression coherence, and the prevalence of protein-protein interactions. The purple/green bar in (**a**) represents the number of TFs that have higher functional enrichment score in our/random result. The purple/green bar in (**b**) represents the number of TFs that have higher expression coherence in our/random result. The purple/green bar in (**c**) represents the number of TFs that have the prevalence of protein-protein interactions in our/random result.

Our result is extracted from Reimand et al.’s result by removing the predicted false positives, which are about 10% of Reimand et al.’s result. Although we have shown (in the Results section) that our result is better than Reimand et al.’s result, it would be more convincing if we can also show that our result is better than the random results. The random result was obtained by randomly removing 10% of Reimand et al.’s result. By repeating this process ten times, we acquired ten random results. We then compared our result with these ten random results by assessing the functional enrichment, the expression coherence, and the prevalence of protein-protein interactions. As shown in Figure 5, our result outperforms all these ten random results, suggesting that our result is of statistical significance.

### Our method performs better than two other existing methods

Two other methods have been developed to infer TF knockout targets using He et al.’s TFKMs. The first method, developed by Hu et al. [8], defined the TF knockout targets by using an error model for identifying differentially expressed genes. The second method, developed by Jiang et al. [30], refined Hu et al.’s result using the TF binding and gene expression similarity information provided by ChIP-chip data and gene expression data, respectively. Since these two methods aim to solve the same biological problem as our method does, the performance comparison should be done. We compared our result with the results of Hu et al.’s and Jiang et al.’s by assessing the functional enrichment, the expression coherence, and the prevalence of protein-protein interactions. As shown in Table 2, our result is more biologically meaningful than their results, suggesting that our method is better than these two existing methods in identifying high-confidence TF knockout targets (see Additional file 8: Table S7 and Additional file 9: Table S8 for details).

**Table 2:** Our result is more biologically meaningful than Hu et al.’s and Jiang et al.’s results

**Test (with FDR = 0.05)**	**Our result vs. Hu et al.’s result**	**Our result vs. Jiang et al.’s result**
Functional enrichment	Our result has an equal or higher enrichment score than Hu et al.’s result in 83% (93/112) of the cases.	Our result has an equal or higher enrichment score than Jiang et al.’s result in 83% (29/35) of the cases.
Expression coherence	Of the 112 TFs, 86% (96/112) show significantly higher expression coherence in our result, compared with only 2% (2/112) in Hu et al.’s result.	Of the 35 TFs, 49% (17/35) show significantly higher expression coherence in our result, compared with only 31% (11/35) in Jiang et al.’s result.
The prevalence of protein-protein interactions	Of the 112 TFs, 82% (92/112) are enriched for membership to a protein-protein interaction module in our result, compared with only 38% (43/112) in Hu et al.’s result.	Of the 35 TFs, 91% (32/35) are enriched for membership to a protein-protein interaction module in our result, compared with only 31% (11/35) in Jiang et al.’s result.

To show that our result is more biologically meaningful than Hu et al.’s and Jiang et al.’s results, we assessed the functional enrichment, the expression coherence, and the prevalence of protein-protein interactions. Our result outperforms their results across all measures. Note that the comparison between our result and Jiang et al.’s result was performed on 35 TFs since only the TF knockout targets of these 35 TFs were reported in Jiang et al.’s study.

### Two issues related to our method are discussed

Two issues related to our method are worthy of discussion. First, there is tradeoff between coverage and precision for using different underlying network to search the possible paths from a knocked-out TF to its knockout targets. We tested two underlying networks. The first one was the TF binding network whose edges are supported only by TF binding evidence deposited in the YEASTRACT database [31]. The other one was the TF regulatory network whose edges are supported by both TF binding and TF regulation evidence deposited in the YEASTRACT database. Our analyses showed that using the TF binding network as the underlying network, the coverage (i.e. the percentage of the biologically interpretable knockout targets) is 90% but the precision (i.e. the average confidence score of an identified path) is only 18%. The confidence score of a path is defined as the ratio of the TF-gene pairs (along the direction of the identified path) that has literature evidence of TF regulation (see Additional file 2: Table S1 for more details). On the contrary, using the TF regulatory network as the underlying network, the coverage reduces to 23% but the precision increases to 73%.

The low precision (18%) resulting from using the TF binding network is not surprising since the overlap between the TF binding data and TF knockout data is very low. Several possible reasons have been proposed in the literature [8,32] to explain this low overlap. First, only a subset of bound TFs may affect a target gene’s expression, depending on the location and orientation of binding sites and the presence of other cofactors [8]. Second, different TFs occupying the same promoter could compensate for each other’s loss, masking the deletion effect [8,32]. Third, a TF could bind a promoter under normal growth conditions but function under other specific stressful conditions [8]. On the other hand, the low coverage (23%) resulting from using the TF regulatory network is also understandable. The reason is that TF regulation information with experimental evidence in the literature now is not rich enough to construct a biologically meaningful TF regulatory network. That is, there are too many missing edges (i.e., false negatives) in the constructed TF regulatory network. We believe that this problem will be solved in the near future since the high-throughput experimental technology for systems biology study evolves rapidly.

The other issue is about the predicted false positives. Our method regards an original TF knockout target as a false positive if no path (in the TF binding network) from the knocked-out TF to this target could be found. However, the knocked-out TF may regulate some of its knockout targets through TF-TF interactions at the protein level but not through transcriptional regulatory chains. In that case, our method would incorrectly regard a real TF knockout target as a false positive. We investigated the severity of this problem in details. Among the false positives defined by our method, only 4% (153/3492) has independent literature evidence of TF regulation other than Reimand et al.’s study [11] (see Additional file 10: Table S9 for more details). Therefore, we believe that most of the predicted false positives indeed represent the noises in the original TF knockout targets.

## Conclusions

In this paper, we developed a method that can extract biologically interpretable TF knockout targets from the original TF knockout targets inferred solely from the noisy TFKMs. An original TF knockout target is said to be biologically interpretable if a path could be identified from the knocked-out TF to this target in the TF binding network. Our refined TF knockout targets outperform the original TF knockout targets across all measures: the functional enrichment, the expression coherence, and the prevalence of protein-protein interactions. Moreover, the identified paths from the knocked-out TF to its knockout targets in the TF binding network form experimentally testable hypotheses of how a TF may regulate its knockout targets. About seven hundred hypotheses generated by our method have been experimentally validated in the literature. We believe that the other hypotheses provide valuable information for biologists to design traditional gene-specific experiments for studying the molecular mechanisms of gene regulation.

## Methods

### Data sources

Two data sources were used in this study. First, the original TF knockout targets of 112 TFs under study were downloaded from Reimand et al.’s study [11]. The knockout targets of each TF (in Reimand et al.’s study) were those differentially expressed genes identified by applying the moderated eBayes *t*-test [12] to Hu et al.’s TFKMs [8]. Second, the ChIP-chip data used to construct the TF binding network were downloaded from the YEASTRACT database [31]. This is the most comprehensive ChIP-chip dataset since almost all the ChIP-chip data available in the public domain are collected in the YEASTRACT database.

### Finding a shortest path from the knocked-out TF to its knockout targets in the TF binding network

In our method, an original TF knockout target (inferred solely from the noisy TFKMs) is said to be biologically interpretable if a path from the knocked-out TF to this target could be identified in the TF binding network. A famous graph search algorithm, called the breadth-first search (BFS) algorithm [33], in the graph theory was modified to search paths in a network. Our modified version can handle loops in the graph which cannot be done in the original BFS algorithm. For each original TF knockout target, our modified BFS (mBFS) algorithm was applied to find a shortest path from the knocked-out TF to this target in the TF binding network. The pseudocode of our mBFS algorithm is as follows.

mBFS (Directed graph = TF binding network, Start node = Knocked-out TF, Destination node = TF knockout target being tested):

INITIAL STAGE:

Set Visited_list and Waiting_list be two empty sets.

Add Start node into Waiting_list.

Set Path[Start node] = Start node.

Set Path[i] be an empty set for each node i (except for Start node) in Directed graph.

LOOPING STAGE:

**while** (Waiting_list is not empty)

{

 Remove the first node v in Waiting_list.

 Add v to the end of Visited_list.

** for** (each direct successor u of v in Direct graph)

 {

 **if** (u is the Destination node)

 {

   Add u to the end of Visited_list.

   Append u to path[v] and set it path[u].

*   TERMINATE while loop.*

  }

**  else if** (u is not in Visited_list)

  {

   Add u to the end of Waiting_list.

   Append u to path[v] and set it path[u].

  }

 }

}

OUTPUT STAGE:

  **if** (path[Destination node] is empty)

   **return** “No Path Exists!”

  **else**

   **return** path[Destination node]

### Calculating statistical significance using the hypergeometric distribution

The hypergeometric distribution [34] was used to calculate the statistical significance of the TF knockout targets for being in a protein-protein interaction module. The details are as follows. Let *S* be the set of the TF knockout targets, *T* be the constructed protein-protein interaction module according to Reimand et al.’s definition [11],* V* =* S* ∩ *T* be the set of the TF knockout targets that are also in the constructed protein-protein interaction module, and *G* be the set of all genes in the yeast genome. Then the *p*-value for rejecting the null hypothesis (H_0_: the TF knockout targets are not enriched for the membership to a protein-protein interaction module) is calculated by

(1) $$p=P\left(x\geq \left|V\right|\right)=\sum_{x\geq \left|V\right|}^{\min\left(\left|S\right|,\left|T\right|\right)}\frac{\left(\begin{array}{c} \left|S\right|\\ x \end{array}\right)\left(\begin{array}{c} \left|G\right|-\left|S\right|\\ \left|G\right|-x \end{array}\right)}{\left(\begin{array}{c} \left|G\right|\\ \left|T\right| \end{array}\right)}\text{,}$$

where |*G*| means the number of genes in set *G*.

## Competing interests

The authors declare that they have no competing interests.

## Authors’ contributions

WSW conceived the research topic and provided essential guidance. WSW and THY developed the algorithm and wrote the manuscript. THY performed all the simulations. Both authors have read and approved the final manuscript.

## Supplementary Material

Additional file 1 **Figure S1.**Provides the detailed explanation of why we only reported the analyses results of 112 TFs. Click here for file

Additional file 2 **Table S1.**Provides the numbers and the detailed gene lists of our refined (i.e., biologically interpretable) and the original TF knockout targets identified by Reimand et al. for the 112 TFs under study. Moreover, the identified path for each biologically interpretable TF knockout target can also be found in this table. The identified paths form experimentally testable hypotheses regarding the molecular mechanisms of how a TF may regulate its knockout targets. Click here for file

Additional file 3 **Table S2.**Provides the detailed information about the functional enrichment test of the refined and the original datasets. Click here for file

Additional file 4 **Table S3.**Provides the detailed information about the expression coherence test of the refined and the original datasets. Click here for file

Additional file 5 **Table S4.**Provides the detailed information about the protein-protein interaction enrichment test of the refined and the original datasets. Click here for file

Additional file 6 **Table S5.**Provides the detailed information about the 692 hypotheses (generated by our method) that have been experimentally validated in the literature [35]. Click here for file

Additional file 7 **Table S6.**Provides the detailed information about the tests of the functional enrichment, expression coherence, and the prevalence of protein-protein interactions of the signals and the noises. Click here for file

Additional file 8 **Table S7.**Provides the detailed information about the tests of the functional enrichment, the expression coherence, and the prevalence of protein-protein interactions of our result and Hu et al.’s result. Click here for file

Additional file 9 **Table S8.**Provides the detailed information about the tests of the functional enrichment, the expression coherence, and the prevalence of protein-protein interactions of our result and Jiang et al.’s result. Click here for file

Additional file 10 **Table S9.**Provides the detailed information of those TF knockout targets that were predicted (by our method) as false positives but have independent TF regulation evidence in the literature [35] other than Reimand et al.’s study. Click here for file
