# Supplementary material for: Identifying biologically interpretable transcription factor knockout targets by jointly analyzing the transcription factor knockout microarray and the ChIP-chip data
Source: BMC Syst Biol. 2012 Aug 16;6:102. doi: 10.1186/1752-0509-6-102 (PMC3465233; doi:10.1186/1752-0509-6-102)

Supplementary Figure 1 - Only 112 TFs have 'enough' ChIP-chip data for our analyses

After analyzing Reimand et al.'s TF knockout targets of 269 TFs, we found two distinct clusters. The first cluster consisted of 112 TFs whose percentages of biologically interpretable knockout targets are greater than 80%. The other cluster consisted of 157 TFs whose percentages of biologically interpretable knockout targets are less than 5%. We found that the TFs in the second cluster have only few or even no ChIP-chip data in the YEASTRACT database. That is, the low percentages of the biologically interpretable knockout targets of the TFs in the second cluster result from the lack of the ChIP-chip data. Therefore, we said that only the 112 TFs in the first cluster have 'enough' ChIP-chip data for our analyses. All the results presented in the paper were based on the analyses of these 112 TFs.

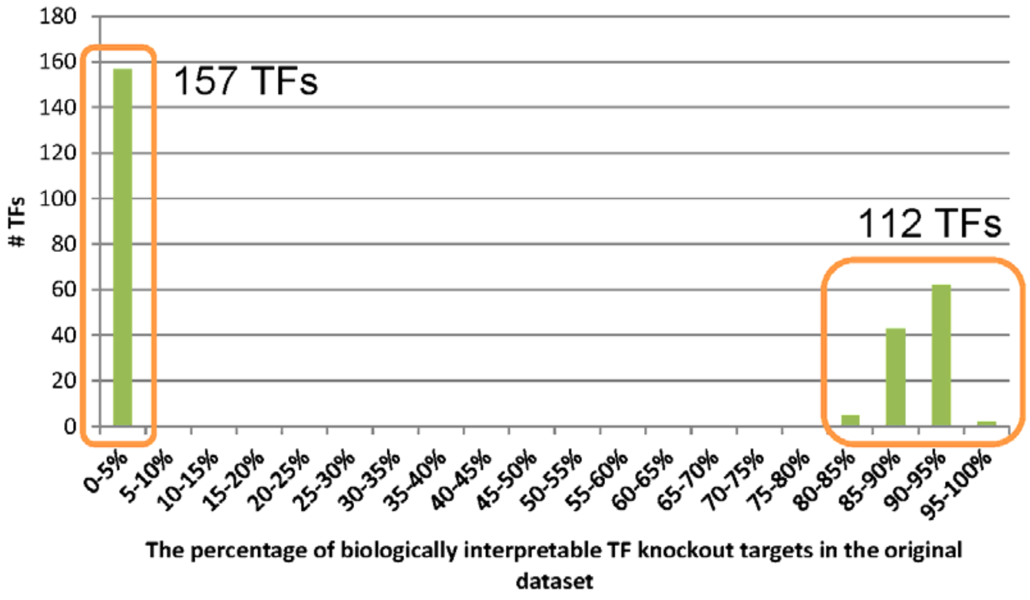

Supplement: Additional file 1 — Figure S1.Provides the detailed explanation of why we only reported the analyses results of 112 TFs. [file 1752-0509-6-102-S1.pdf]
